# Response to “Critical Assessment of the Evidence for Striped Nanoparticles”

**DOI:** 10.1371/journal.pone.0135594

**Published:** 2015-11-10

**Authors:** Quy Khac Ong, Francesco Stellacci

**Affiliations:** Institute of Materials, Ecole Polytechnique Fédérale de Lausanne, Lausanne, Switzerland; aker Hughes, SAUDI ARABIA

## Abstract

Stirling et al., (10.1371/journal.pone.0108482) presented an analysis on some of our publications on the formation of stripe-like domains on mixed-ligand coated gold nanoparticles. The authors shed doubts on some of our results however no valid argument is provided against what we have shown since our first publication: scanning tunneling microscopy (STM) images of striped nanoparticles show stripe-like domains that are independent of imaging parameters and in particular of imaging speed. We have consistently ruled out the presence of artifacts by comparing sets of images acquired at different tip speeds, finding invariance of the stipe-like domains. Stirling and co-workers incorrectly analyzed this key control, using a different microscope and imaging conditions that do not compare to ours. We show here data proving that our approach is rigorous. Furthermore, we never solely relied on image analysis to draw our conclusions; we have always used the chemical nature of the particles to assess the veracity of our images. Stirling et al. do not provide any justification for the spacing of the features that we find on nanoparticles: ~1 nm for mixed ligand particles and ~ 0.5 nm for homoligand particles. Hence our two central arguments remain unmodified: independence from imaging parameters and dependence on ligand shell chemical composition. The paper report observations on our STM images; none is a sufficient condition to prove that our images are artifacts. We thoroughly addressed issues related to STM artifacts throughout our microscopy work. Stirling et al. provide guidelines for what they consider good STM images of nanoparticles, such images are indeed present in our literature. They conclude that the evidences we provided to date are insufficient, this is a departure from one of the authors’ previous article which concluded that our images were composed of artifacts. Given that four independent laboratories have reproduced our measurements and that no scientifically rigorous argument is presented to invalidate our STM images, and also given that Stirling et al. do not contest the quality of our recent STM images, we re-affirm that specific binary mixture of ligands spontaneously form features in their ligand shell that we describe as stripe-like domains ~1 nm in width.

## Introduction

Stirling et al. offer a critique of the body of work on striped nanoparticles [1]. In a series of papers in the last ten years we have shown that when some binary mixtures of molecules coat ~ 5 nm gold nanoparticles (NPs) stripe-like domains of alternating composition spontaneously form. Experimental evidences for the existence of these domains come mainly from scanning tunneling microscopy (STM) [2,3,4,5,6,7,8] but are strongly supported by other techniques, such as atomic force microscopy (AFM) [9], nuclear magnetic resonance (NMR) [10], and small angle neutron scattering (SANS) [5]. Extensive modeling and theoretical work on these systems has been done by the Glotzer’s group [11] and recently supported by other groups [12,13,14]. We refer, for example, to ref. [15] for a better summary of these results.

**Fig 1:**
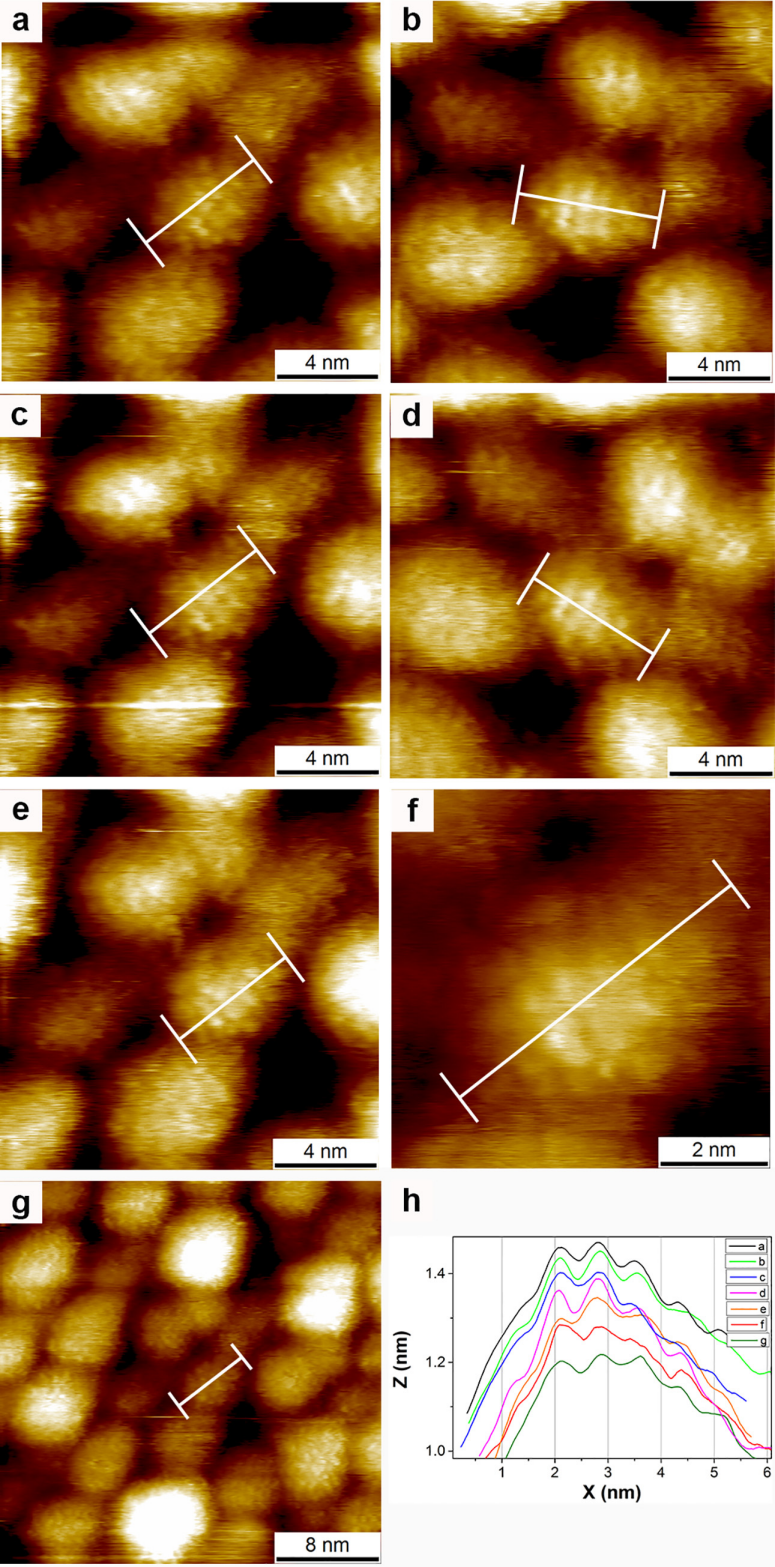
Image reproduced from reference [7] showing topography STM images of a sample of mixed ligand nanoparticles imaged at different scan angles and scan sizes. The cross section achieved by averaging the images at the white band shown on them is presented in (h). All images are show in their full scan range. A complete description of the images is found in reference [7]. Reproduced from reference [7]. Reproduced by permission of The Royal Society of Chemistry.

The STM evidences for the existence of stripe-like domains on nanoparticles are based on images that show elongated features (that we refer to as ‘stripe-like domains’) on top of nanoparticles coated with a binary mixture of ligands (see Fig 1 as an example). The characteristic spacing of these domains is ~1 nm. When homoligand nanoparticles are imaged features with ~0.5 nm spacing appear. At high resolution these features are resolved as dots, at lower resolution they appear somewhat as stripe-like domains. Dots [16] and stripes [17,18] on homoligand alkanethiol-coated NPs at ~0.5 nm spacing have been observed in other laboratories. Also in the case of ~1 nm stripe-like domains, as the resolution of the images is increased it is possible to observe these domains break-up into dots spaced ~0.5 along the domains and ~1 nm across them, further confirming that the imaged domains are formed by densely packed ligand molecules. A complete discussion of the reasons that lead to dots merging intro stripes can be found in references [6,7], and has been an underlying theme in our literature as we have always implied that stripe-like domains are due to images of dots that merge together.

Starting from our very first publication [4] we stated that STM artifacts needed to be distinguished from stripes on nanoparticles: “*Particular care was used in assuring that the observed nanostructures were not imaging artefacts…*”. As a consequence we analyzed noise on gold hemi-spheres, flat surfaces, and nanoparticles to compare it to images of striped nanoparticles. We found that -in the range of gains and imaging parameters we used- noise features show a spacing that scales linearly with tip-speed. On the contrary, features on mixed-ligand nanoparticles imaged within the same imaging-parameter space show minimal dependence on imaging speed. The dependence (or lack of) of the features on imaging speed has always been the key discriminator we have used to distinguish real features from artifacts [2]. Simulations of STM feedback loop when imaging under feedback-loop oscillations have confirmed our interpretation [2,15]. Also, starting from our first publication we have shown that the width of the domains we observe on nanoparticles depended on the chemical composition of the ligand shell.

Recently, we have imaged the same stripe NP sample in three different laboratories (plus our own laboratory, so four in total), and sent old and new images to be analyzed in another laboratory [15]. All four laboratories have identified tip-speed invariant stripe-like features on the same sample with the same spacing. We have used a newly developed power spectral density (PSD) analysis to prove that all of the features on the nanoparticles have the same characteristic length and are independent of scan size, scan speed, imaging parameter, laboratory, microscope, and operator [15]. In addition to this stringent reproducibility test, we have achieved also high-resolution images in which single molecules aligning to form stripe-like domains are indeed visible [6,7]. The PSD analysis has also confirmed that homoligand nanoparticles’ STM image show features on the particles with a correlations length that is roughly half that found on mixed ligand particles.

**Fig 2:**
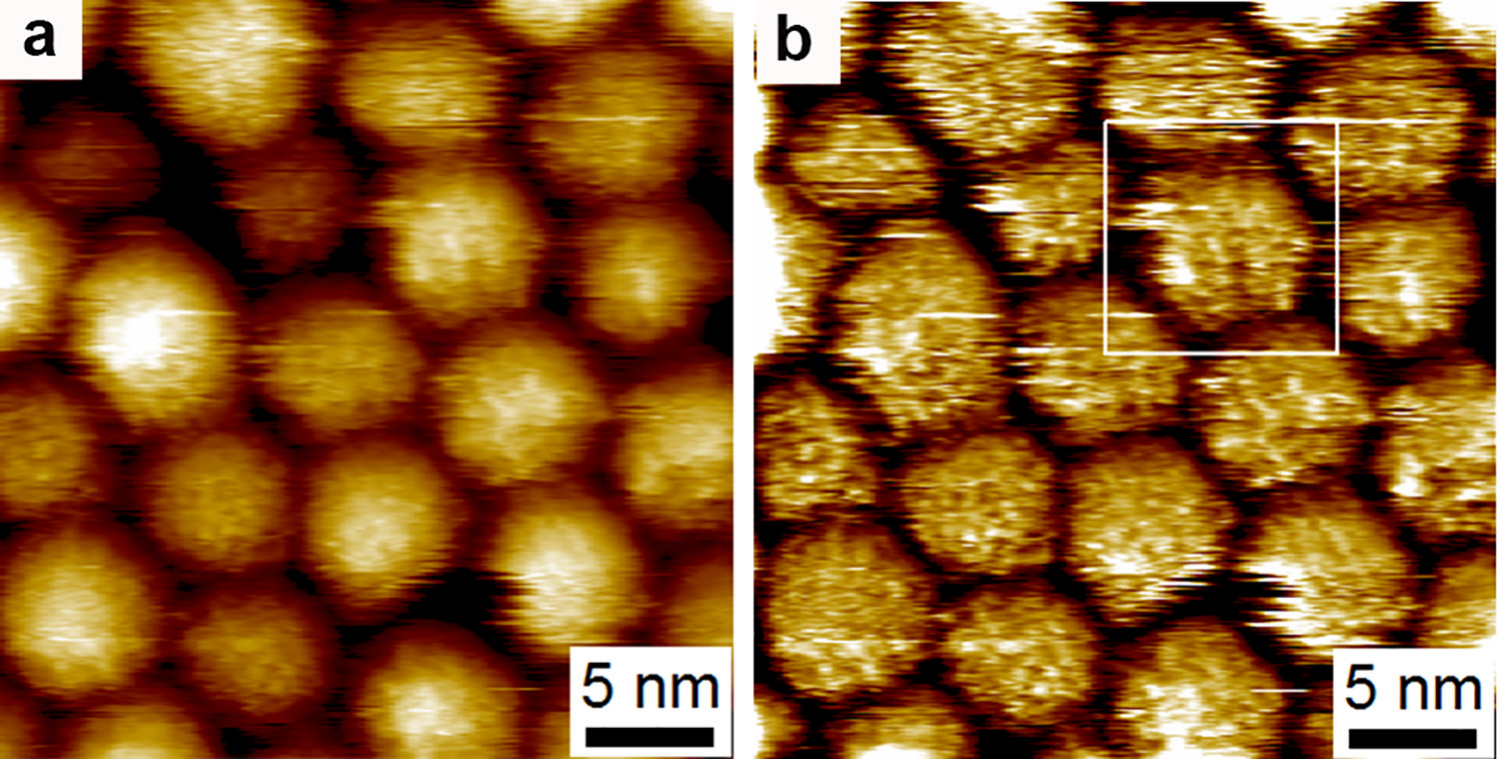
(a) STM image of a sample of nanoparticles imaged in phenyl octane. This image is taken from reference [5] (Moglianetti et al. Scanning Tunneling Microscopy and Small Angle Neutron Scattering Study of Mixed Monolayer Protected Gold Nanoparticles in Organic Solvents. Chem. Sci. 2014, 5, 1232–1240; http://dx.doi.org/10.1039/C3SC52595C) where a full description of the image and of the sample can be found. Reproduced by permission of the Royal Society of Chemistry. All rights reserved. (b) Same image as the one shown in (a) with curvature removed using the same procedure described in reference [1], this is done so to highlight the features on the nanoparticles it should be noted the alignment of these features across many particles.

In reference [1] our results are critically discussed by using miscellaneous arguments. Hereafter we show that all of them are either technically incorrect or inconclusive. The paper dedicates two out of eighteen pages to analyze our first image, while briefly it comments (in one case positively) on the new images. We would like to start our reply in a positive tone, pointing out to the images and conclusions where we could all agree. The authors of reference [1] state “*Thus*, *to reliably verify the existence of specific topographic structure it is important to systematically probe the features by comparing the trace and retrace images from the STM*, *taking repeat scans of the same feature*, *rotating the scan direction*, *deliberately modifying the tip in order to ascertain the level of tip-sample convolution*, *and zooming in on specific features in ‘real time’*, *i*.*e*. *by reducing the scan area imaged by the STM*, *to check that features are unchanged*.” Indeed, in our recent efforts to image mixed-ligand nanoparticles we have obtained images that fulfill all of these criteria. The images presented in reference [7] have clear stripe-like features on nanoparticles, that are invariant with (i) imaging speed (all other parameters kept constant), (iii) scan direction (i.e. trace and retrace match very well), (ii) scan angle, (iii) imaging parameters, and with features that are conserved (within reason) on the nanoparticles across multiple scans. Fig 1 here reproduces these images; in reference [7] we also prove that substantially identical features can be imaged with different tips, ruling out conclusively any possible effect of tip-imaging as opposed to nanoparticle-imaging. Fig 2 reproduces images published in reference [5] of the same quality as the ones shown in Fig 1, differing only in the contrast between the two ligands, given that in the first case (Fig 1) the particles are coated with a mixture of aromatic and aliphatic ligands, and in the second (Fig 2) both ligands are aliphatic. We should point out that the authors of ref. [1] recognize the value of the images shown in Fig 2 saying “*Although Fig. 1 of Moglianetti et al. shows arguably the most convincing images of nanoparticle sub-structure we have seen to date in the work of Stellacci and co-authors (the persistence of features in the trace and retrace images is particularly compelling)*”. The only critique present in reference [1] to the images in Fig 2 is that the interpretation of stripe-like domains on the nanoparticles is arbitrary. We will leave to the readers to interpret whether or not this is the case, Figs 1 and 2 are there to be judged. To us, these images confirm our position: in STM images, features on nanoparticles are due to true tip-sample interaction when they are imaging-speed invariant and have characteristic dimensions that scale with the chemical composition of the nanoparticles. To the authors of reference [1] these images may be the first to fulfill all of the requests of controls in scanning probe. Whatever the case, the scientific conclusion is that mixed ligand nanoparticle present stripe-like domains ~1 nm in width.

During the refereeing process for this paper, the authors of reference [2] have stated that they believe that issues of tip convolutions “However, in our discussion of the liquid images (Moglianetti et al., reference 29 in our paper) we discuss tip convolution effects as a possible cause of these features.” are present in the images shown in Fig 2. In fact, we have no record of this in reference [1], yet this objection is easy to overturn as (1) all particles in the images shown in Fig 2 present different features, that they conserve upon rotation hence it would require a very special tip to keep being imaged in different ways for different nanoparticles, (2) images like the ones in Fig 2 are shown in Fig 1, these have the same scientific message than the ones in Fig 1 but are achieved with different tips, also in reference [7] we have shown PSD analysis from at least 20 images, these where acquired with at least 5 tips, it would be a truly unique feature if all of these tips had 1 nm features on them (while all of the tips used to imaged homoligand nanoparticles had ~0.5 nm features).

Hereafter we summarize the development of this debate.

## State of the Debate

In 2012, Levy and his team published a paper aimed at critiquing our results [19]. The paper disputed the STM results by stating that (i) the spacing of the domains we show in our image is inconsistent with a projection of a curved surface onto a flat imaging plane and (ii) that the 2D spectral density (commonly called Fourier transform in literature) of the images we presented was inconsistent with a random alignment of the stripes. The first argument (i) is in direct contradiction with scanning probe microscopy literature of images of carbon nanotubes and of viruses [20,21]. We have also specifically addressed it in reference [6]. The second argument -at most- would show that stripe-like domains in our images are aligned (a feature still present in our recent images where the alignment rotates with scan angle, see both Figs 1 and 2 here), and as discussed in reference [8] the data are shown without proper controls. Now, Levy as one of the authors of reference [19] together with a new team has written a new paper on our work (reference [1]), which re-analyzes for more than half of its length the same images analyzed in 2012 (reference [19]), but does not refer to the arguments used in the past, and this time reaches the conclusion that our images are not conclusive.

There is only one thing that these two papers have in common: they state that we describe particles coated with stripe-like domains as having a high degree of order in their ligand shell; it is our feeling that this is a misrepresentation of our work. Since 2012 we have consistently represented the nature of the domains that form on nanoparticles as only locally ordered, calling them ‘stripe-like domains’. In particular in the reply to Levy’s Small paper (reference [19]) we wrote: *“In 2007*, *in a theory paper supported by experimental evidence*, *coarse grain and molecular dynamics simulations of our particles were presented*. *The simulations showed the presence of stripe-like domains with local order*. *Since then we have used in all of our publications a definition of our particles as containing “stripe-like” domains*. *We have since found a good number of structure–property relationships that link this particular configuration of ligands with macroscopic properties*. *None of these properties require a ‘perfect’ stripe alignment but only a local one*. *Representing our work as ‘perfect’ stripes on nanoparticles is a misrepresentation*, *and we hope that this communication will help to clarify this point*.*”*

The authors of reference [1] decide to ignore completely this detailed clarification based on the theoretical understanding of this problem that we gained in 2007 and published in reference [11] and that we have cited in any of our papers. On the contrary, in reference [1] the authors still say repeatedly “*do not provide compelling evidence for the highly-ordered striped morphology claimed by Stellacci and co-workers*.” and “*the highly-ordered stripe pattern which Stellacci et al*. *have repeatedly put forward*” and *the very regularly spaced*, *well-aligned striped features claimed by Stellacci et al*. *throughout their work”*

Furthermore we want to make the reader fully aware that in our literature we had dealt with scanning probe artifacts, starting from the caption of Figure 1 in our very first paper [4]. We have also addressed it extensively in our second paper [2] and in all of our subsequent microscopy papers. Indeed as we will show here all of the issues raised in the paper were already dealt with in our literature better than what was described in reference [1].

It should be pointed out that in no point of reference [1] the analysis presented provides sufficient condition to prove that evidences in our papers are wrong. Instead, what is given is only a long list of circumstantial observations to cast doubts on the body of our literature. In reference [1] we find little and confused criticism to our data, and in fact the new images recorded in our and in other laboratory are simply described as not showing stripes. This unilateral description is in complete disagreement with our and other STM expert groups’ description and interpretation of the images.

## Discussion

We respond here to all of the technical issues raised in reference [1] trying to be concise and to maintain the order of presentation of such paper, even though as stated in the past science is a process where understanding develops with time, hence in a debate it would definitively be more correct to analyze newer and hence more developed images and concepts first, and then older and hence less complete ones later.

### Issue of high and negative current

The author of reference [1] note that in our old images current ranges from positive to negative values, concluding (in their paper and more strongly in their referee report) that this is unphysical and hence it must derive from the saturation of the preamplifier in our STM. We note that they provide no experimental image to support this conclusion, neither a simulation on an electronic circuit, so the logic on which this is based is hidden to the reader. More importantly, as previously provided to them, we show in **Fig A in S1 File**, that in any Bruker microscopes, when images are saved with off-line planefit option turned on, current images do contain both positive and negative values as a result of this mathematical operation. We have conclusively proven this point by saving consecutive images one right after the other with and without off-line planefit. We get identical current images, except that with the off-line planefit on the current values span from a negative value that is dictated by the mathematical plane-fitting operation. This is general for a multitude of imaging conditions and samples we have tested. Hence this specific argument is totally incorrect. We should point out that in our images, historically, as we stopped using off-line planefit option, we stopped observing current ranges that varied from positive to negative values.

**Fig 3:**
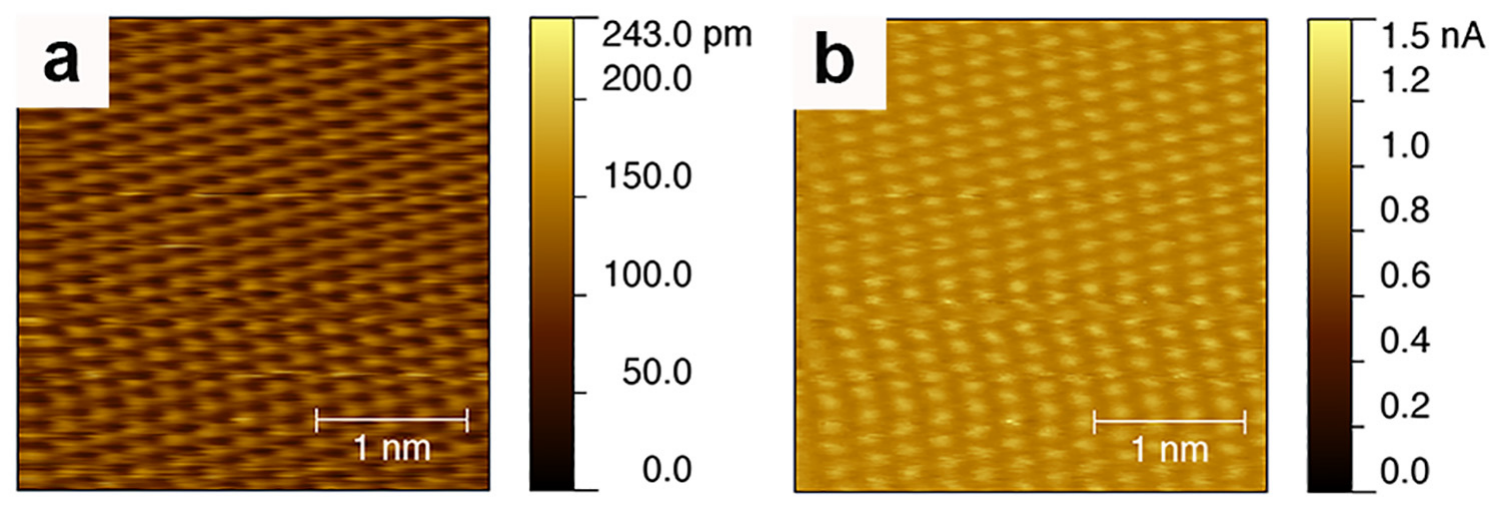
STM topography (a) and current (b) image of a sample of highly oriented pyrolitic graphite (HOPG). The tracking of the topography and current image is clear. The scanning parameters are the following: current setpoint 1.0 nA, bias voltage 80 mA, integral gain 1, tip velocity 0.18 μm/s. The image is consisted with the large literature of STM images of HOPG [22].

It is also pointed out that the current range in one of our images is very large (118.2 nA vs. current setpoint of 0.84 nA). The significance of this observation is unclear. First we should point out that this observation is limited to a single image. Through the years we have presented many more images consistent with that one image (in terms of stripe-like domains) that have significantly lower currents, for example (0.32 nA vs. current setpoint of 0.05 nA) in 2014 [7]. More importantly, the range of current in an image of a complex sample such as ours does not in itself mean anything unless it is shown as a histogram. The argument in reference [1] is that large currents are due to constant error correction and hence feedback loop oscillations, this argument holds if current values in the images are high for most of the image, this information is not contained in a single number as the current range. This is because in an STM current image there are always a few pixels that have a large current value for a variety of reasons. In **Fig B in S1 File** we present histograms for the current values of the 2004 image in question and of two more recent images, as well as for the graphite [22] image shown in Fig 3. In all cases most of the image has current around the setpoint (as it should be) with a few pixels that inevitably have large values. These histograms do not reveal any major difference between the images.

In Figure 1 (d) of reference [1] the authors flatten the very image they dedicate a significant part of their paper to show how the topography image is similar to the current image. The implicit conclusion from this analysis is that the similarity is due to ringing. This conclusion is incorrect, as the similarity (tracking) between topography and current images may be a necessary condition for ringing in scanning probe images, but for sure it is not a sufficient condition to establish ringing. Indeed, in Fig 3 we show an example image of graphite with a large current range and perfect tracking of the topography and the current image.

We restate that 2004 images showed stripe-like domains on NP with widths that were independent of scan speed as analyzed back then and reanalyzed in 2013 using a PSD analysis [15].

### Issue of pixelation

The authors of reference [1] indicate that some of our original images are digital zooms of larger images. They refer to the few number of points present in the scan lines to determine the presence of stripes and to measure their width. We emphasize that never in our literature have we drawn conclusions on measurements of features above the Nyquist frequency. Indeed the measurements taken through the years have been confirmed in another laboratory by a PSD analysis [15], and their values are not disputed in reference [1], hence this discussion does not affect any scientific conclusion in our papers. In reference [1] there is a statement that our measurements are based on a few pixels, and hence somehow they should be less reliable. This statement is questionable on many grounds. First this is not the case for all of the images we used already in 2004, second, the conclusion drawn in such papers in terms of spacing of stripe-like domains being ~1 nm are confirmed by high resolution images as the ones shown in Fig 1 of this paper that contain many more pixed, and can be extracted with PSD analysis.

### Issue of alignment

**Fig 4:**
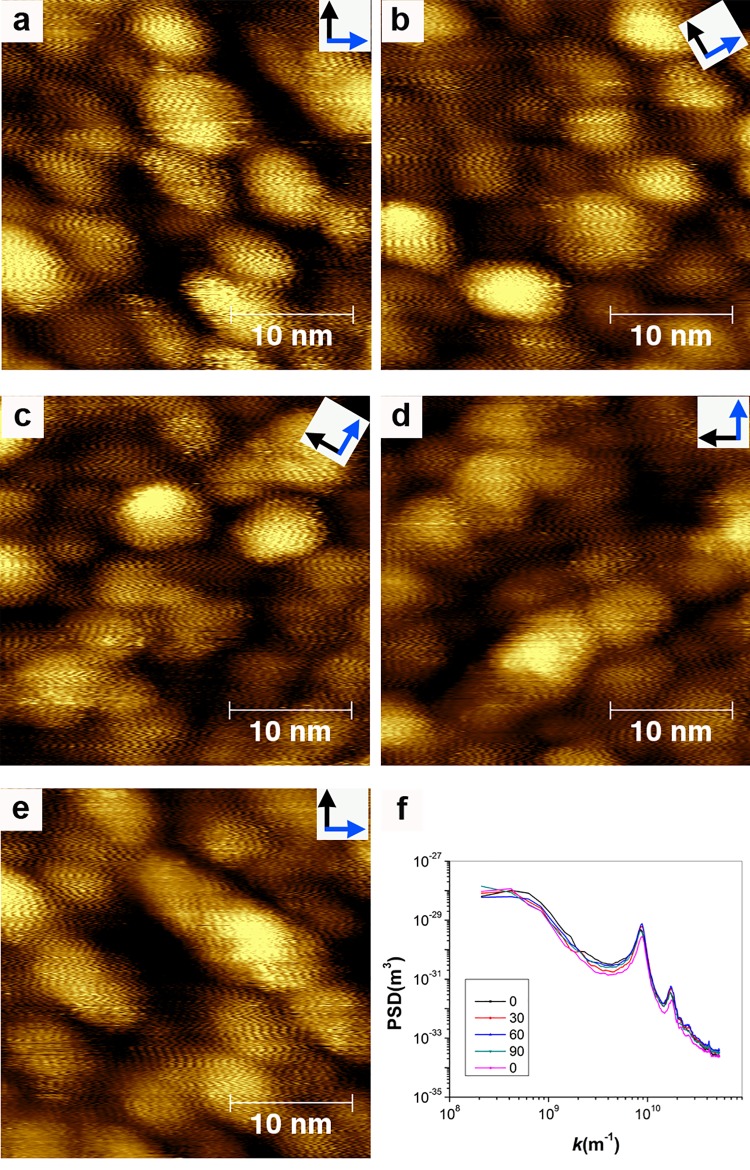
Topography STM images obtained by inducing feedback loop oscillation on a sample of nanoparticles. Scan angle is varied across the images that the features do not change direction. (f) 1D PSD plot of the images showing sharp peaks at the spatial frequency corresponding the spacing of the feedback loop oscillation (larger peak) and to its first harmonic.

The authors describe the alignment of stripes (again in the same single image they dedicate two out of the eighteen pages of their paper) as a problem in our images. The authors claim that alignment over the scan area present is not physically possible but present no backing for their statement. First we point out that alignment of features is not a proof of feedback loop artifacts as it is something that can be present in a sample (one could image a grating with perfectly aligned features). In Fig 1 we show conclusively that particles align their stripes when close to each other and that such alignment rotates with scan angle, as opposed to the lack of rotation that can be seen for feedback artifacts (Fig 4). The same alignment is also evident (over a larger area) in Fig 2. Hence, we prove that alignment is indeed a characteristic of our sample.

### Issue of stripes going from our particle into another

The authors claim that stripes in a single image of ours appear to go from one particle into the next one. This again is not present in most of the images that they asked us to put online, and definitively not in our recent ones. Importantly they authors overlook the fact that such image has a low resolution, (even though they describe such low resolution in paragraph *“The distances measured range from approximately 2 to 4 pixels and thus are very close to the (Nyquist) resolution limit…”* in Stirling et al.) and that few pixels are present between the stripes, thus making the apparent continuation of the stripes a simple effect of the pixelation.

### Issue of feedback gain settings

**Fig 5:**
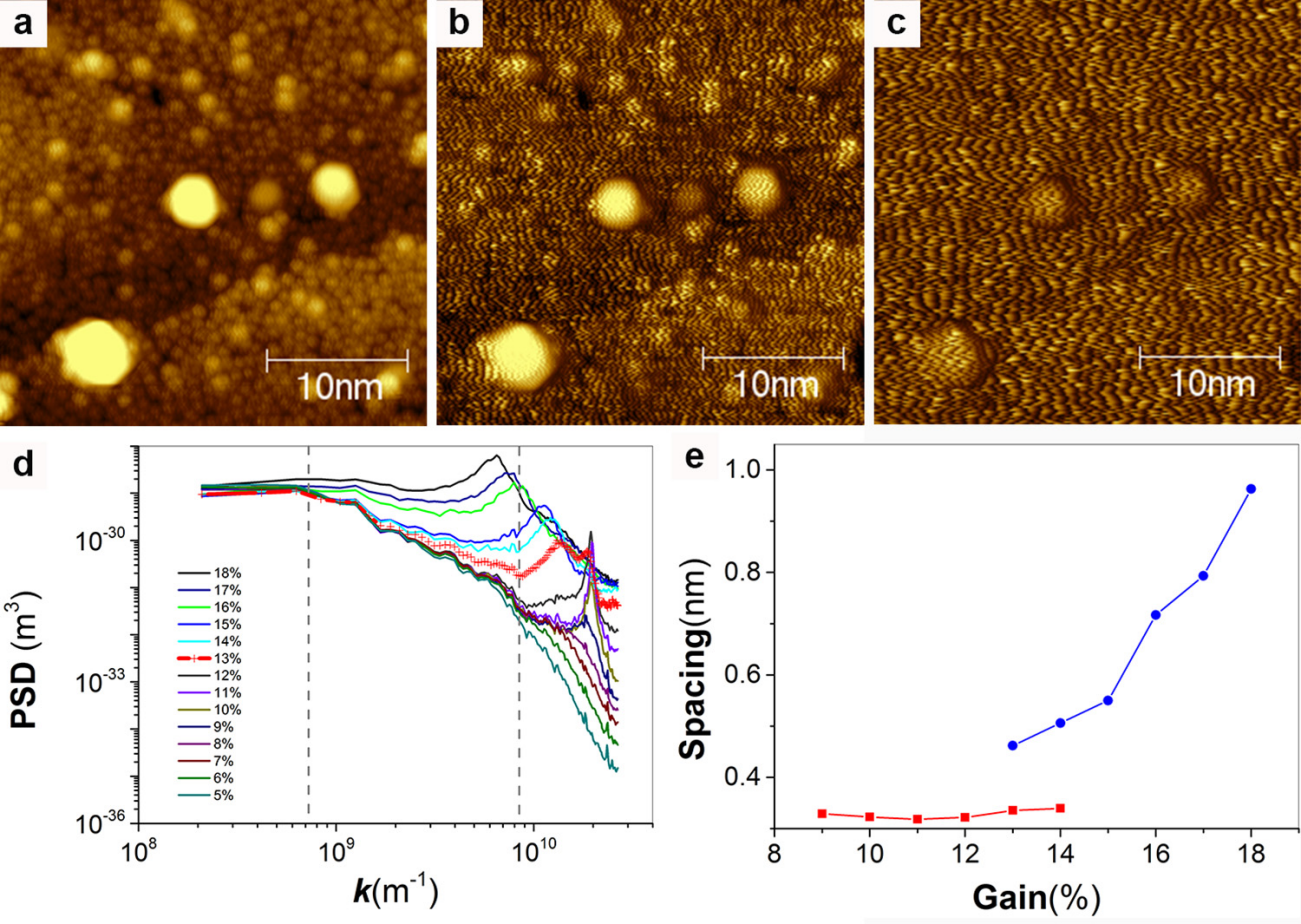
(a to c) STM topography images used in reference [1] and downloaded from http://dx.doi.org/10.6084/m9.figshare.882904 on August 11, 2014. Insets of these images are used in reference [1] Fig 3 to argue that gains affect the spacing for feedback loop artifacts. The images shown are three representative ones over a set of 10. (d) PSD plot for all 10 images. Peaks for the feedback loop oscillation are clearly visible. Plots for images at 13% (red) and 14% (light blue) show two peaks. The image at 13% is the image shown in (b). Image in (a) is a representative image from the group below 13% and the one in (c) comes from the group above. In (e) we plot the trend for the spacing corresponding to the frequency at the peak in the PSD plot for these images, in red are all of the peaks for the curves below 13% and for the lower spacing peak for the two curves at 13 and 14%. In blue the peaks for the higher spacing for these two images, and for single peak in the images acquired at higher gains. In (d) the dotted lines demark a region in the PSD with many features corresponding to the sizes of the hemispheres present in the sample. As long as these features are present (gains below 13%) one can assume that the tip is tracking the sample. In this case the feedback loop oscillations all happen at the same spacing, above 14% tracking is lost (as evident when looking at the PSD) and the oscillations start becoming dependent on the gains. Evidently at 13 and 14% the imaging happens in a mode that is somewhat in between the two. The analysis of this data shows that what stated in reference [1] is incorrect.

**Fig 6:**
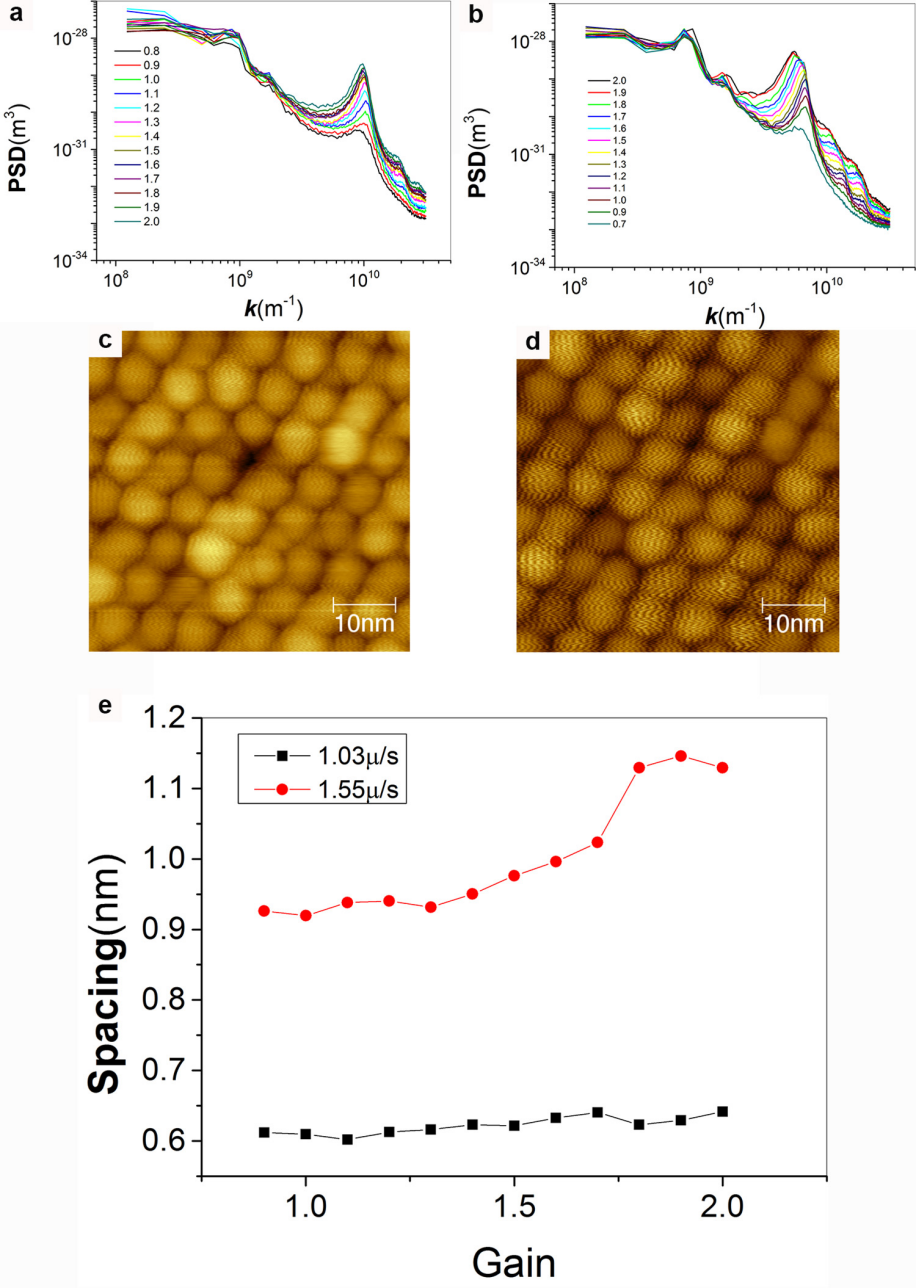
(a and b) 1D PSD plots for STM topography images of nanoparticles imaged with feedback loop oscillations induced. Plots in (a) are for images at 1.03 μm/s and in (b) are at 1.55 μm/s. (c and d) representative images for the images analyzed in (b) taken with the integral gain of 1 and 1.8, respectively. (e) Plot for the trend for the spacing corresponding to the frequency at the main peak in the PSD plots shown in (a, black) and (b, red). In the case of (a) we see minimal dependence on gain; in (b) we observe a weak dependence after the gain of 1.5, where the PSD plot show a start of loss of tracking. In any case the change in spacing measured is only ~ 0.2 nm (20% of the smallest spacing measured at this speed). We should highlight that the vast majority of our images are at speeds below 1.0 μm/s and are obtained currently at gains below 0.5 while historically at gains below 1, hence is a regime where in our microscope there is no dependence of the measured spacing on gains.

In reference [1] two figures (Figures 2 and 3) are presented to show that variations in gains can produce artifacts due to feedback-loop oscillations that appear as stripes on nanoparticles. First, we reiterate that in our literature we have extensively discussed this point and presented to the reader plenty of artifact images referring to the similarities between the images of stripe-like domains. Second and most importantly, the authors of reference [1] are arguing that gains in STM change the period of oscillation in feedback loop artifact and consequently can be used to offset the characteristic linear dependence that this period has over imaging velocity. This concept is technically wrong. As shown in Fig 5, proper analysis of the experimental images presented in reference [1] performed either by PSD (but one can also reach the same conclusions using manual measurements) shows that feedback loop artifacts have an almost constant period as long as the tip tracks the sample, when tracking is lost then the period depends on the magnitude of the gains. In Fig 6 we show that in our microscope for the most part gains do not vary the oscillation period in feedback loop artifacts. In a single case ringing could be pushed toward losing tracking of the sample and then a weak dependence appeared. Given that in all of our images we are always tracking the sample we can confidently state that we have always worked in a range of gains that does not affect the spacing of the observed features neither for artifact nor for real features, hence it remains that the main discriminant between these two regimes is the speed dependence of the features. This indeed can be seen comparing the values for feedback loop oscillation at the two speed analyzed in Fig 6. In conclusion, this point, the sole technical critique to our approach in the whole paper, is incorrect.

We should add that using a different microscope to test the response of feedback loop artifacts is incorrect as the feedback noise affects the appearance and quality of STM images in ways that vary according to the specific design of the STM. For example, the response of the piezo scanners, the designs of its feedback electronic circuit, the sensitivity and stability of the instrument are just a few important factors that vary from microscope to microscope. Simply looking at images without providing any proper analysis and generalizing effects to all microscopes one incurs in gross mistakes as the ones that we have highlighted in reference [1].

### Issue of measurements in JACS paper

In Figure 4 of reference [1] and in its discussion Levy and coworkers try to re-analyze spacing measurements performed and illustrated in Figure 3 of our 2006 JACS paper [3]. This figure was meant to illustrate how noise and real features can be present on the same image and can look very similar. Yet, these features scale differently with scan velocity. We should point out that this image and its analysis are in a tutorial part of the paper where we are indeed discussing noise. All of the conclusions in the paper are achieved by analyzing other images that are indeed recorded at higher magnification and with minimal noise. Yet in reference [1] the authors analyze the whole image, and show that variations in features dimension is such images is quite large. As expected our measurements (both for real features and for artifacts) all fall within the range they determine. Their analysis applies to whole image while the analysis we present was based exactly on the part of the image shown in such Figure 4 of reference [3]. Importantly, that image was and is meant to show the similarities between noise and real features for our image, something the authors of reference [1] present in their paper as a new concept. All of the conclusion and measurements shown in reference [3] are not based on that set of images.

### Summary discussion on old images

In conclusion the re-analysis of our old images by Levy and co-workers is based on many observations of features in the images (high current, pixelation, alignment) that cannot be uniquely associated with noise. Their sole technical point on our analysis (based on the independence of the features observed on imaging speed) fails in its premise, as they claim that this independence can be due to varying gains in our images but within the (very narrow) gain range that we have always used there is no dependence of ringing period on gains. This independence is present also in the very images that they chose to show from their own microscope (when the sample is tracked). Additionally, gains can have very different effects on different microscopes and consequently a comparison across microscopes is questionable.

In our original work we extensively show that spacing of features in our particles depends on their coating, illustrating how homoligand particles (or close to homoligand particles) have a spacing of ~0.5 nm as opposed to ~1 nm for mixed ligands. This important feature is not discussed in the reference [1].

After having failed to show that our original images are due to feedback oscillation, the authors of reference [1] attempt to attribute a variety of other artifacts to our more recent images, where according to their own parameters they find no evidence of feedback oscillation.

### Issue of random noise

In reference [1] the images presented in our own 2012 Small paper [8] are analyzed by summation to show that the image so achieved has no stripe feature. The claim is that random noise on the images forms stripe-like domains, and that this random noise can be cancelled by summing all the images. First, it is known that ligand molecules on gold particles are mobile to some extent [23,24] and hence one cannot expect that features are invariant for an indefinite amount of time. In many images published recently we have shown how features stay for many images, hence this is indeed a feature of our imaging, but these images have to be taken consecutively so to minimize the time in between images. Negligently, the authors sum up the images taken non-consecutively and hence with an unknown time delay, and also images taken at different scan angles. The latter is known to be problematic as the low pixelation of the images (that they underline) does not allow for an easy interpolation of the image points (necessary to rotate a scanning probe image). Both these operations are problematic if not incorrect. If we sum up the trace and retrace images of the two consecutive images recorded we find stripes to be present there (see **Fig C in S1 File**) at a resolution that is comparable between a single image and the sum of four images, indeed also the 1D PSD of these images show the same feature in the 1 nm region. This finding and our most recent images (see Figs 1 and 2), convincingly show the persistence of stripe-like features on nanoparticles for subsequent images. Hence the argument in reference [1] is incorrectly presented.

### Interpretation of high-resolution images

We have recently presented high-resolution images of some particles [6]. In particular in Figure 2 of reference [6] we show single dots (i.e. end groups of single molecules) that align to form stripes with a characteristic spacing of ~1 nm across the lines and of ~0.5 nm along them. In reference [1] the authors suggest that these dots could have a random distribution. They present no mathematical support to their suggestion, and cannot dispute the distances and distance distribution we measure. We leave to the reader the interpretation of the image that we reshow in **Fig D in S1 File** with relevant distances highlighted. The authors of reference [1] also suggest a completely different problem with our images. They claim that, for these high-resolution images, it is possible that the tip was been imaged by our sample. For this latter as well as for the previous suggestion the authors of reference [1] neglect that the high-resolution images presented were recorded on samples that had been imaged at lower resolution with the same or with other tips (and in one case in other laboratories) and in all case a characteristic spacing of ~1 nm was found. This completely invalidates their arguments.

**Fig 7:**
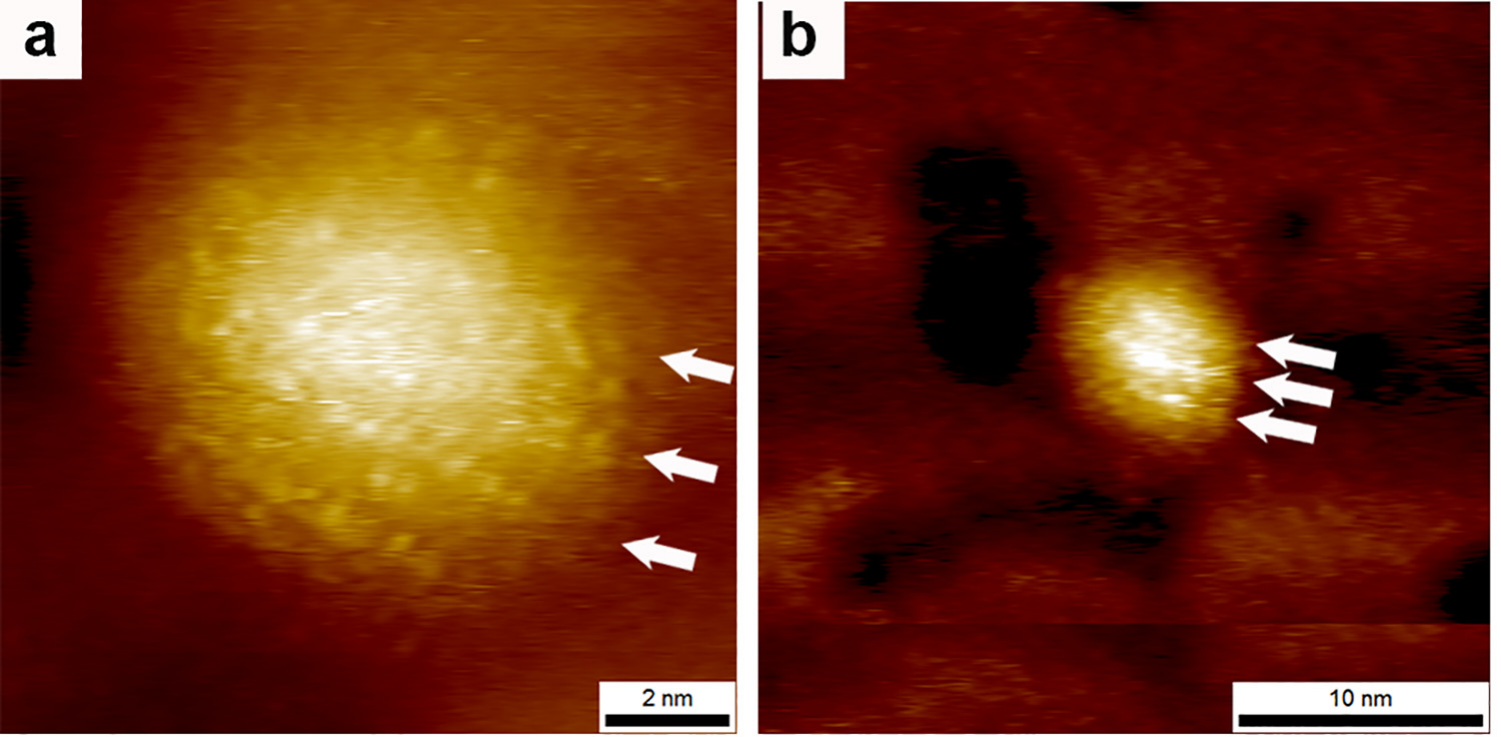
STM topography (reproduced from reference [6]) images of the same nanoparticles imaged at higher and lower resolution the arrows indicated the persistence of the same three stripe-like domains as the scan size is varied. Importantly resolution decreases the dots present in (a) merge into continuous lines in (b). Reprinted with permission by the American Chemical Society.

As for the evolution from high-resolution dot-like images to low-resolution stripe images we have specifically addressed this point in Figure 3 and S10 of reference [6] where we show the continuous change of the former into the latter upon zooming out, here in Fig 7 we re-illustrate this concept.

This argument is presented by taking a single image out of its content so to make a convenient argument while neglecting all of the rest of the work done to validate such image.

### Imaging of Janus particles

In reference [1] it is stated that images of Janus particles can be due to tip morphology. We are aware of this problem and for this reason we have shown an image with only a few particles being Janus and the ‘Janus line’ being in various orientations. This cannot be explained by the data presented in reference [1]. Hence their argument, while correct on its own, does not apply to our images and our particles.

### Biscarini et al. Langmuir

At this point we would like to summarize the content of our recent paper [15], as we believe that this paper is strongly misrepresented in reference [1]. In this paper, we first describe the imaging of a single sample performed in four different laboratories (including ours) on five microscopes. The images are all compared to each other (and to one original 2004 image) in Fig 1 of that paper and described as “*Fig 1 provides qualitative evidence that similarities exist in the images acquired at the different laboratories*”. Hence in all laboratories (as in 2004) striped-like domains spaced ~1 nm were identified. In order to measure (not identify) the characteristic length scale of the features present on the particles we developed a whole new method to analyze these images using 1D PSD plot. The result of the PSD analysis on the images achieved were that all laboratories had imaged features on the particles with the same correlation length, and that these features where invariant on imaging parameters, microscopes, or laboratory. When the PSD analysis was applied on homoligand nanoparticles it was found that the correlation length of the features present on the nanoparticles was approximately half that found from mixed ligand particles (i.e. ~0.5 nm), indicating a clear dependence of the features present on the images on the chemical composition of the nanoparticles. The same PSD analysis was applied to the 2004 images. It showed that indeed the features imaged showed a correlation length of ~1 nm and that this correlation length was independent of imaging parameters. The analysis was extended to noise and it confirmed that in such case features had a correlation length that scaled linearly with tip speed (independent of gains and other parameters). Thus the PSD analysis confirmed the assumption that we have made since 2004 on the dependence of imaged features on imaging speed.

Stirling et al. present a critique to this paper that is technically incorrect, hereafter we reply to all of the points raised.

#### Quality of the images

The first critique to the Langmuir paper is that images of our particles taken in different laboratories are free of artifacts but show no visible stripes. This is in disagreement with what all of the authors state in Biscarini et al. Moreover, in reference [1] while critiquing the same paper (ref. [15]) the authors state that PSD analysis is not correct for STM images of striped nanoparticles as for example when analyzing their own simulated images they say: “*We also note that the stripes are clearly visible to the eye before the 1D PSD peak becomes noticeable*.” So they claim that in their images stripes are there even when a PSD peak is not pronounced yet (something we agree with and we prefer to err on that side) and yet for our images that show a pronounced PSD peak they also claim to not be able to see stripes. Importantly this statement contradicts what experts in all laboratories state they were able to see in the images “*Fig 1 provides qualitative evidence that similarities exist in the images acquired at the different laboratories…”*, what scan lines show and what PSD measures.

The contrast in the images shown is not the highest because molecules with similar chemical nature but differing only in length were imaged. We chose these molecules as we were concerned with cleanness of the sample in a long storage time (needed to allow four different laboratories to image the same sample).

#### Use and significance of the PSD analysis

Importantly in reference [15] it is stated that PSD cannot be used to determine whether there are stripes in our images, because PSD plots do not distinguish between different shapes of features. Reference [1] misrepresents our work. Never in our work do we use PSD to identify stripes. We use PSD to quantify objectively the characteristic correlation lengths present in our images, and determine unequivocally that such lengths do not vary with (a) imaging parameters (among them tip velocity), (b) imaging laboratory and/or microscopy, and (c) imaging operator. Images were used to determine the presence of stripe-like domains, and PSD to quantify their characteristic length scale.

In reference [1], the main critique to the new images is that PSD might be quantifying ‘randomly positioned speckles’ in our images, as illustrated in Figure 10 of reference [1]. Yet there is no explanation for why these ‘randomly positioned speckles’ should be invariant with imaging speed, with imaging laboratory, but should vary in characteristic length scale upon the change in ligand shell composition from 1 nm in mixed ligand particles to 0.5 nm in homoligand particles. Are there ‘randomly positioned speckles’ that can distinguish between mixed and homoligand nanoparticles? The invariance of the PSD features with imaging parameters (for example scan size) conclusively excludes that the PSD is showing features that are artifact of the image itself (e.g. pixelation), implying that the PSD feature are due to true features on the particles. Such features are reproduced across laboratories. At this point two main geometries can lead to such PSD, dots roughly 1 nm in diameter or elongated features with a 1.0 nm spacing. The latter we call stripe-like domains. The former we can conclusively state has never been observed in our images. At times we do observe dot 0.5 nm in spacing in images of homoligand nanoparticles.

We conclusively state that their hypothesis is inconsistent with our data. In references [5,6,7,15] it is shown that PSD of NP images substantially overlap in spatial frequency. When re-plotted in time frequency these PSD separate, showing that the features present on the images (whose characteristic length scale is captured by the PSD) are independent of imaging speed. This excludes that the PSD is identifying random scars on the images.

Finally there is, in reference [1], a critique to our fitting approach. We will not discuss this in detail here, as it is irrelevant to the discussion (characteristic correlation lengths can be extracted manually if one wishes to, or one can simply extract a range of distances from the relevant feature in the PSD plot without fitting the plot, as Levy et al. did in reference [19]). We should point out that the result of the fitting performed on simulated images as the ones shown in Figure 7 of reference [15] are in quantitative agreement with the length scales of those images (known exactly as it was imposed at the time of simulation). And that the critique that a seven-parameter fit cannot be brought to fit a curve is peculiar to say the least. In the referee report, the authors of reference [1] claim that their point of the fit is that “a) A seven-parameter non-linear fit is easily biased by initial conditions leading it to converge to a different answer. b) That during the analysis certain areas were excluded without this being mentioned in the original work. c) That when we applied these same fits we got poor convergence warnings.” The latter point (c), is exactly the point we made above, we did not get these warning when performing the fits, the readers can judge. As for point (b) some regions where excluded only for low quality old images, the PSD of the new images the main point of reference [15] were fully analyzed, also some region were excluded for high resolution images, but in this case the spacing on the images are easier read by direct measurements and the results of the PSD matched this measurement precisely. As for point (a), as shown in reference [15] and more recently in reference [7] the PSD analysis leads to the same results of direct line-profile measurements, hence the bias in the analysis can be excluded.

### Images in liquid

In liquid we have achieved images that have received praise from the authors of reference [1] but have been critiqued as showing features on nanoparticles whose interpretation is biased. We first start by stating that images like the one in Fig 2 in this paper show features whose molecular nature we and the authors of reference [1] all agree upon. Such images have a 1D PSD in complete agreement with the discussion in reference [15], again invalidating the discussion in reference [1] about such argument. The only disagreement on those images is that supposedly there is bias in interpreting these features as stripe-like domains. Let’s clarify that as specified above we do not believe that stripes on nanoparticles are highly ordered and have made this clear in multiple occasions for example in our recent reply to one of the authors’ paper [8]. We present features on particles as stripe-like domains, such features are elongated features with a characteristic correlation length of ~ 1 nm, this is exactly what can be seen in Fig 2 and in all other images presented in references [3,4]. If we all agree that in such images there are molecular features, and given that the characteristic correlation length of this features is ~1nm (however measured), then how can this not be described as stripe-like domains? Nowhere in the images there are dots (or dot-like domains), the elongation (and alignment) is evident.

### Summary discussion on newer images

In conclusion the critique to the newer images presented in reference [1] is severely affected by a selective choice of images and concepts, but does not hold when compared to all of the data we presented.

### Other techniques

We discuss here the critique to the other techniques used to establish our work.

#### NMR

Levy and co-workers present a peculiar critique to our NMR analysis of nanoparticles. It can be summarized as such. There are other (typically more complex) approaches to model NMR signal for patchy particles beyond what we have presented in reference [10]. For example, we can start from the critique to the linear dependence of NMR shift for randomly distributed ligand arrangement. They show that such dependence could also be non-linear. Yet our experimental data is linear and matches our model. Hence it is very difficult to understand the purpose of their discussion. Even if some random distribution could lead to non-linear dependences (something one would need to experimentally prove), our particles were designed to have a random distribution, showed no evidence of patchiness in the STM images, and had a linear dependence of the NMR signal whose only valid explanation is that of a random distribution of ligands.

In reference [1] we find the usual list of issues (line widths, other peaks that could have been studied, etc.) that *could* have been problematic in our measurement but no pinpointing to these problems is present. The experimental evidence is that the peak position for the aryl proton resonance does have a linear shift that can be assessed rigorously.

As for the model of the 1/x dependence for Janus particles a similar complex argument is developed, where a more complex model is made to fit better our data while reaching the same conclusions, that is that particles are indeed Janus. Importantly in this case such conclusion can be reached simply by looking at the 2D NMR spectra, in agreement with the literature. Our model solely confirms this finding.

As for the sinusoidal dependence of the shifts (that we attribute to stripe particles) they develop alternative patchy models to explain such trends. This model has to assume patchy particles but changes the patchiness geometry. It remains unclear whether such model can explain the presented 2D NMR data. Importantly though, the authors fail to point out that the trends presented in our paper for Janus and striped particles are captured on particles of *identical* composition differing *only* in size. The size dependent change from Janus to stripe particles is consistent with the theoretical explanation we have given for Janus/striped particles, while it remains completely unexplained in their alternative explanation. Hence, the presented discussion is solely an alternative approach to interpret our data, it agrees with all of our conclusions but with the form of the patches in striped nanoparticles, yet it provides no explanation to one of the central experimental evidences of our paper.

#### SANS

The authors claim that the ab-initio calculated structure is *“in stark contrast to (and could in fact be considered strong evidence against) the very regularly spaced*, *well-aligned striped features claimed by Stellacci et al*. *throughout their work”*. In other words the only critique to our work is that it does not support high order in striped nanoparticles. Yet we never claim any high degree of order we only claim stripe-like domains with a certain correlation length. Hence the very basis of their discussion (SANS not being proof for highly ordered stripes) stems from a misrepresentation of our work, as we do not claim such order. Equally misrepresented is the characteristic length scale that we extract from the SANS measurement. We do find a spacing of 1 nm and a spacing of 1.5 nm (caption of Figure 6 in reference [5]), on the nanoparticles we find a spacing of 1.1 nm (specifically referring to a plateau in the PSD plot from 1 nm to 1.6 nm). Of course one can compare the latter spacing with any of the two spacings extracted from SANS (or with an average of the two), but to say that “*STM PSD differs by 50%”* is a clear misrepresentation

Moreover, we do not believe that the discussion presented is correct. First as one does SANS in liquid, the expect scattering from a sample with more or less degree of order in the stripes -at the wave-vectors that we study- is expected to be practically identical (for example due to the random particle rotation during the scattering). Hence, an ab-initio simulation approach (such as the one we use, MONSA) would produce the same fitting independent of such order. The main thing one can extract by such measurement is a correlation length of the features present and such correlation length we have estimated in the paper to be ~ 1 nm in great agreement with the correlation length in the STM images (that the authors of reference [1] do not contest). Hence, these two very different techniques find the same information on our particles.

#### Simulations

Reference [1] ends with a discussion of the many simulations that have established stripes on nanoparticles. On one hand, the simplicity of the model used is critiqued, specifically the fact that the complexity of the thiol-gold bond is lacking. Yet the extensive work done in this field by the groups of Glotzer [11], Cacciuto [13], Egorov [12], Edlund [14], Aleksander-Katz [25], shows a general phenomenon based on the balance between entropy and enthalpy. No explanation is provided for why a complex yet mobile thiol-gold bond would hinder such a general phenomenon based on entropy/enthalpy arguments. Another critique is the fact that in some simulations ligands are not allowed to leave the surface, we point out that the only effect of this phenomenon is either the quicker reaching of equilibrium (i.e. stripes) or the effective lowering of the ligand density. The latter has been found to have little effect on the final morphology.

Importantly, in reference [1] it is claimed that simulations were done to support experiments. This is incorrect, as nothing in the simulations was done to bias the result. It remains unquestionable that many different simulation codes (from coarse grained, to self-consistent field, to full atomistic) have all found stripes. The excellent matching between experiment and simulations cannot be denied.

## Conclusions

In conclusion, the authors or reference [1] keep systematically misrepresenting our data. They continuously portray our model of striped nanoparticles as a highly ordered one, against what we have been saying in our literature and what–for example- we have stated in a recent reply to another paper of one of the authors of reference [1]. To foster a healthy debate we have tried to highlight in this paper the images that could represent the basis for a dialogue. When addressing one of our latest papers (reference [6]) the authors themselves comment positively on the images, but they conclude that the images do not show clear stripes *“there is strong observer bias in the identification of “stripes”*. The nature of this bias is not specified. These images show a clear characteristic length scale of 1 nm and clear nanoparticles substructure. A representative of such image is shown here in Fig 2, to allow the reader visualize and decide on this issue. Images like the one in Fig 1 or Fig 2 are free of any of the problems that are claimed in reference [1]: they show what we have been saying in the last 10 years, that mixed ligand nanoparticles have stripe-like domains ~1 nm in width. Extensive simulation literature provides an explanation for the phenomenon. When homoligand nanoparticles are imaged ~0.5 nm features are present in our and many others measurements. In some cases these features blur into stripes exactly as imaged ligands do in striped particles. This phenomenon we have addressed with experiments in our literature.

In this paper we have shown that all of the arguments of reference [1] are either inconclusive, or technically incorrect (see for example the case of the effects of gains on feedback loop artifacts), or in direct contradiction with the data presented. We have also demonstrated that the controls that we have done throughout our literature are indeed correct and resist the harshest critics. Our images present features on mixed ligand nanoparticles whose characteristic correlation length (~1 nm) is independent of imaging parameters (as well as of imaging microscope, laboratory, or operator) but depends critically on the chemical composition of the ligand shell of the nanoparticles. We are positive that our conclusions will received more and more support as more groups enter this debate and bring their own expertise in this field.

## Supporting Information

S1 File Addititional STM images and futher analyses of STM images. STM images of gold nanoparticles coated with dodecanethiol (**Fig A**). Histograms of current images for STM images (**Fig B**). STM topography image by summation of trace and retrace of an image for the set of images displayed in Figure 4 of reference [8] (**Fig C**). (a) STM topography image from Figure 2 of reference [6] (**Fig D**).
(DOCX)
