# Supplementary figures and images for: Response to “Critical Assessment of the Evidence for Striped Nanoparticles”
Source: PLoS One. 2015 Nov 10;10(11):e0135594. doi: 10.1371/journal.pone.0135594 (PMC4640849; doi:10.1371/journal.pone.0135594)

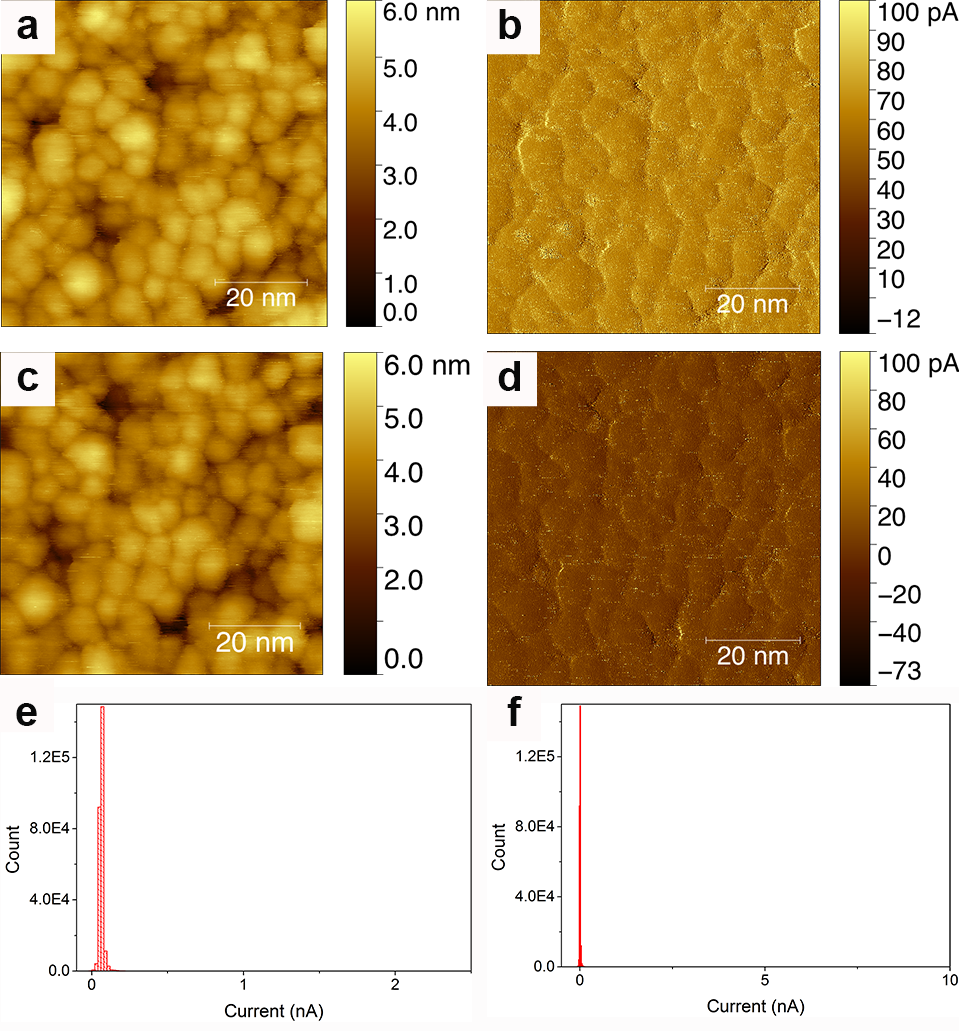


**S1 Fig. A**


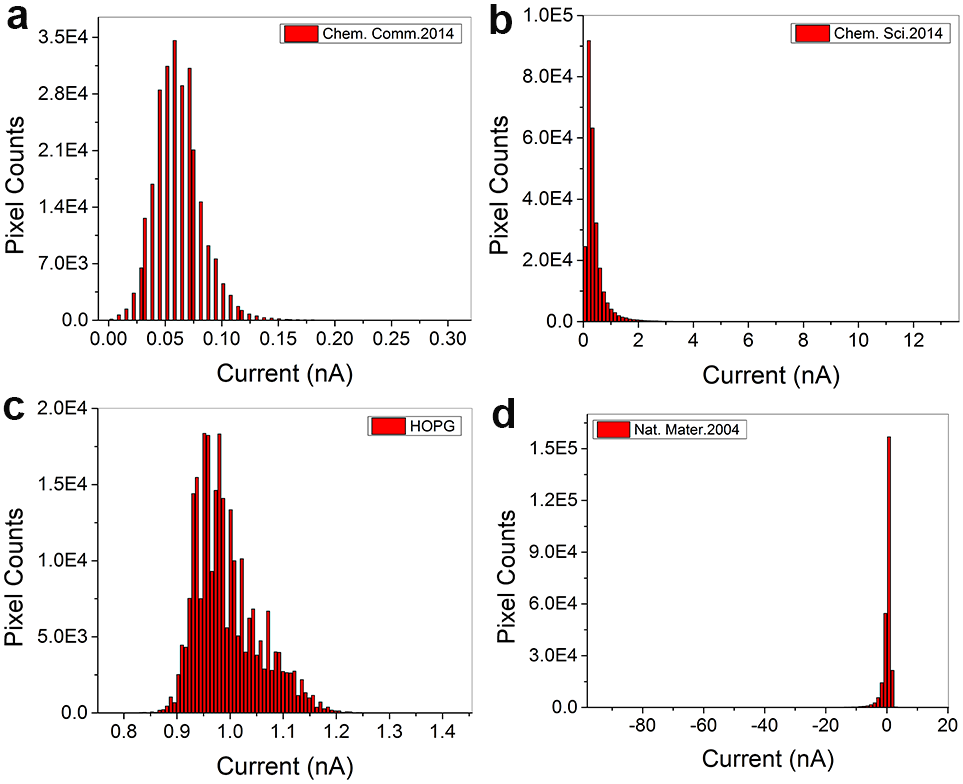


**S1 Fig. B**


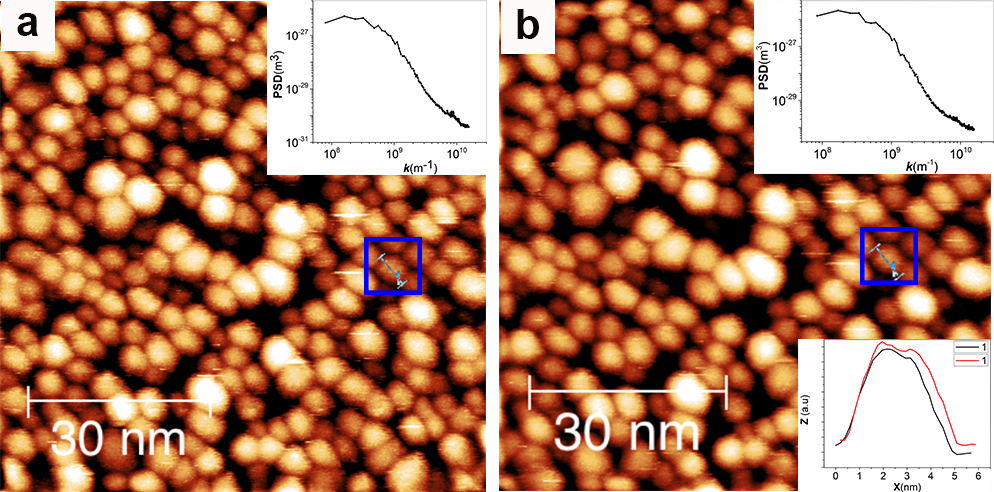


**S1 Fig. C**


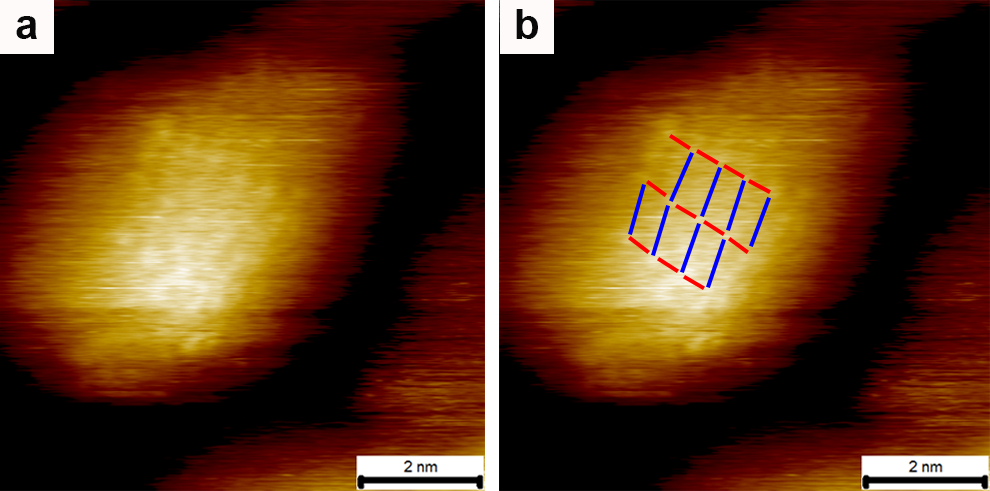


**S1 Fig. D**

Supplement: S1 File — STM images of gold nanoparticles coated with dodecanethiol (Fig A). Histograms of current images for STM images (Fig B). STM topography image by summation of trace and retrace of an image for the set of images displayed in Figure 4 of reference [8] (Fig C). (a) STM topography image from Figure 2 of reference [6] (Fig D). (DOCX) [file pone.0135594.s001.docx]
